# Analysis of capability, motivation and opportunity to prevent substance abuse in sensation seeking students on the outskirts of a city in Eastern Iran: a qualitative study

**DOI:** 10.1186/s40359-022-00748-1

**Published:** 2022-02-22

**Authors:** Seyed Saeed Mazloomy Mahmoodabad, Hassan Zareei Mahmoodabadi, Hssein Mazhari Majd, Mohammad Reza Miri

**Affiliations:** 1 Department of Health Education and Promotion, Social Determinants of Health Research Center, School of Public Health, Shahid Sadoughi University of Medical Sciences, Yazd, Iran; 2 Department of Psychology, Yazd University, Yazd, Iran; 3 Department of Public Health, Social Determinants of Health Research Center, Faculty of Health, Birjand University of Medical Sciences, Birjand, Iran

**Keywords:** Substance abuse, Sensation seeking, COM-B model, Adolescent, Qualitative study

## Abstract

**Background:**

Usually, substance abuse begins in adolescence in Iran. Young people who score high on the sensation-seeking trait tend to be more prone to substance abuse and an early experience in using. According to the COM_B model, substance abuse can be observed in situations where a person has the necessary physical and mental abilities, the necessary opportunities, and competing motivations. Therefore the study aimed to deeper understand of capability, motivation, and opportunities of substance abuse behavior to design educational programs.

**Methods:**

This study was conducted based on content analysis approach. Data were collected through a focus group discussion with 18 participants (high school male students) from the outskirts of Birjand, who received a positive score from the Zuckerman Sensation Seeking Questionnaires' summary form. The group discussion was conducted using a guide to semi-structured questions based on COM-B model constructs.

**Results:**

Students' knowledge of substance cognition, side effects, and consequences of addiction were incomplete. Some students believed that substance use was a way to control or vent emotions and that smoking was a sedative, and private spaces and uncrowded public places were physical environments that students cited for engaging in substance use. In the analysis, 24 subcategories and 11 categories were identified for the capability analysis section, 16 subcategories and 7 categories for the motivation section, and 21 subcategories and 6 categories for the opportunity section.

**Conclusions:**

In order to achieve more precise prevention interventions against adolescents' tendency for substance abuse and to have a more significant impact on their behaviors, it is beneficial to study the three identified factors in some of the target individuals before intervention.

## Introduction

Usually, substance abuse begins in adolescence [1]. Therefore, some studies have shown that substance abuse in adolescents is a severe and ever-growing problem [2], with tobacco, alcohol, and marijuana cited as the commonly used addictive primary substance that adolescents try [1].

Substance abuse is also a relatively common problem among Iranian adolescents. According to a report published by the Iranian Ministry of Health, the prevalence of alcohol use among Iranian adolescents (15–18 years old) is 15%, opium use is 3.1%, and ecstasy use is 5.6% [3]. Following the use of these substances, death events in adolescents and young people occur more than in other age groups [4].

The tendency to use a substance is strongly influenced by various factors such as gender, age, psychological factors affecting sensation seeking, and genetic sensitivity. In addition, interpersonal and social influences also affect adolescents' behavioral choices and health outcomes [5].

Based on studies, three categories of individual, family, and social factors have been identified as influential in Iranian adolescents becoming addicted [6]. Individual factors include variables such as adolescents' attitudes toward substance abuse, self-control, social competence, and family and school attachment [7]. Parental disputes, lack of parental support, parental divorce, and emotional deprivation are family factors that affect adolescents' addiction [8]. Also, attitudes toward school, friends' educational status, friends' attitudes toward substances, friends' abstinence from smoking, and friends' addiction are known as social factors of addiction in Iranian adolescents [6].

Studies have described a significant relationship between youth reckless behavior and sensation seeking. Young people who score high on the sensation-seeking trait tend to be more prone to substance abuse and early experience in using than those who have a low score in this field [9]. The trait of sensation seeking is related to biological and mental sensitivity to the motivational effect and enhanced effects of substances and directly correlates with the early onset of substance use and alcohol consumption [10].

In this respect, environmental factors also act as a mediator or moderator [11]. Research has shown that environmental factors influence the onset of substance use [5]. For example, neighborhoods with poor socio-economic conditions are considered high-risk environments. In these environments, parents' relationship with adolescents can be an influential factor in adolescents becoming substance users. Therefore, it is essential to consider the socio-economic environment, the social impact of peer networks, the effects of parental relationships, and the interactions between them as factors that reduce or aggravate the consequences of health behaviors [12].

Substance abuse has been described in several models, but a general behavior model can be helpful to understand addiction better. According to the available texts [13], a model is considered to explain and intervene in the phenomenon of addiction, which includes a full range of concepts of addictive behavioral models. One of the models with this feature is COM_B (capability (C), opportunity (O), motivation (M), behavior (B)) [13]. According to this model, substance abuse can be observed in situations where a person has the necessary physical and mental abilities (such as self-regulatory capacity, ability to learn from punishment, ability to formulate and adhere to personal rules), the necessary opportunities (such as environmental and social guidelines, the availability of alternative sources of reward, the cost of activities), and competing motivations (such as emotional needs, a need for belonging, the anticipation of pleasure or satisfaction, anticipation of relieving cravings, and fear of rejection).

Therefore, this study aimed to more and deeper understand the capability, motivation, and opportunities of substance abuse prevention in adolescents to design educational interventions to prevent substance abuse based on the COM-B model in a group of high school male sensation-seeking students on the outskirts of Birjand in Eastern Iran.

## Methods

Focus group discussion was used as a qualitative approach to analyze capability, motivation, and opportunity to prevent substance abuse in sensation-seeking students. This qualitative research was conducted in one of the male secondary schools on the outskirts of Birjand, in Eastern Iran. A total of 18 subjects who participated in this study were junior high school students from one of the male high schools (Allameh Farzan) in the suburbs of Birjand who received a positive score from the summary form in the Zuckerman Sensation Seeking Questionnaire [14]. Along with the coordination of the school and a phone conversation with the students' parents, subjects were invited to one of the health centers on the outskirts of the city for an interview. Due to the existence of the COVID-19 pandemic at the time of the study, in line with health protocols (wearing a mask, physical distancing, and environmental ventilation), group discussions were held in two 60-min sessions in which 9 students participated in each session. The parents of one of the students refused to attend the interview due to type 1 diabetes.

The group discussion was conducted using a guide to semi-structured questions based on COM-B model constructs from discussions among the researchers and a review of major preliminary studies. The guideline comprised the following three aspects: capability, opportunity, motivation toward substance abuse, consisting of 18 questions. Authors 3 and 4 were present at the interview. Due to the experience of qualitative interviewing in the past, the task of managing the interview session and asking questions was the responsibility of Author No. 4. At the start of each discussion session, the purpose of the research was explained to participants along with the method of discussion, the confidentiality of the information provided, the optionality of participating in the study, and finally, verbal informed consent was obtained. For a more detailed analysis, the discussions were written and recorded (audio) with the verbal permission of the students. Author No. 3 took notes and recorded the interview.

At the end of each discussion session, the data recorded by the researchers was listened to at the earliest opportunity and compared with notes taken. The text was then read several times to gain a deep and accurate understanding and disaggregated into the shortest meaningful units (code). The codes were then classified according to the constructs of the COM-B model.

In this study, data analysis was performed based on qualitative content analysis, the steps of which were as follows:

- Implemented the entire discussion immediately after each discussion
- Studied the whole text for a general understanding of its content
- Determined the units of meaning and the initial codes
- Classified similar primary codes into more comprehensive classes
- Determined the main theme of the classes

In order to achieve the accuracy and validity of the study, the criteria presented by Lincoln and Guba in 1985 [15], namely acceptability, reliability, transferability, and authenticity, were considered by the research group. Spending enough time at all stages of data collection, analysis and interpretation helped the data acceptability. The full text of several coded discussions was also provided to participants to assess the integrity of the data. For this purpose, some participants were asked to compare the codes assigned to the content of the discussion with the original text and express their agreement or disagreement with the researcher's relevant interpretation of their speeches. To assess the reliability of the data, the researchers gathered the information before promptly interpreting after the discussions using the notes during the discussion, plus noted down the non-verbal behaviors of the participants in the margin section of the notes. In order to increase the transferability, all research processes and work done in the study path were prepared in a clear, accurate, and written manner, and the files were stored and maintained to enable others to follow the research path and the characteristics of the study population. Theoretical saturation was judged to have been attained when no new themes or information emerged in subsequent interviews.

Maxqda 10 and Word 2010 softwares were used to analyze the content.

## Results

All of the 18 selected students participated in the focus group discussion. Among them, 6 (33.33%) were seventh-grade students, 5 (27.78%) were eighth-grade and 7 (38.89%) were ninth-grade. The mean age of the participating students was 14 ± 1 years (Table.1).

**Table 1:** Demographic characteristics of the participants (n = 18)

Demographic variables	Count	Percentage
Mean age	14 ± 1	
*Education level*		
Seventh-grade	6	33.33
Eighth-grade	5	27.78
Ninth-grade	7	38.89
Total	18	100

### Capability analysis

Students' knowledge of substance cognition, side effects, and consequences of addiction was incomplete. Although some common types of substances were mentioned, they did not know all the types of substances. The substances mentioned by the students included heroin, opium, caffeine, amphetamines, crystal meth, and cigarettes. The students mentioned some of the cardiac and pulmonary side effects; however, their information regarding side effects and consequences of substance use was incomplete, and some students even considered substance use beneficial.

Participant No. 2: "Drugs excite young people, and there is no harm in using them once. Cigarettes do not make you feel high, but drugs make you high."

Participant No. 5: "Opium smoke is good for laryngeal cancer."

They did not have enough information about the underlying factors of adolescents' substance addiction and did not know precisely how and in what steps people are involved in substance abuse.

Participant No. 1: "People take drugs once and get hooked all the time."

Participant No. 2: "People easily deceive children."

None of the participants mentioned the main underlying factors of adolescents' substance addiction.

Most of the participating students did not know the correct ways to manage stress and to overcome anger.

Participants 6, 13, and 17: "To control our stress, we pretend to be carefree."

Participant No. 14: "I punch the windscreen to control my anger."

Among the students, some mentioned methods such as "threatening to beat" and "physical confrontation" to cut off contact with an inappropriate friend. Some also used "making wrong remarks by friends, cutting off communication, saying NO firmly and directly to express unwillingness to be friends" as a way to cut off communication with an inappropriate friend.

They also cited strategies such as jumping from heights, dangerous maneuvers on motorcycles and bicycles, smashing glass, acting erratically, fighting, and harassing others to vent their emotions.

The participants wanted to be educated on the effects of substance use on health and behavior, the color and shape of substances, and the penalties imposed for substance crimes. They also expressed interest in researching substances and producing an information poster.

Their preferred media for training was television, mobile phones, and textbooks. They also wished to attend substance rehabilitation camps and health centers to receive training and observe the side effects and issues with substances (Table.2).

**Table 2:** Categories and subcategories obtained from capability analysis

Subcategories	Categories
Cognition of substance, cognition the consequences of substance, cognition of substance side effects	Cognition the substances, its side effects and consequences
Imitation, improper dating, lack of personal communication skills	Communication skills during adolescence
Stress stimulus control, get ready to face the stimulus	Stress prevention measures
Mental activities to control stress, physical activity to control stress	Personal actions during stress
Consulting, get help from others	Interpersonal communication when stress occurs
Indirect and non-verbal expression of unwillingness to continue the relationship, indirect and verbal expression of unwillingness to continue the relationship	Indirect announcement of termination of relationship
Direct expression of unwillingness to continue the relationship, threats and violence	Direct announcement of termination of relationship
Awareness raising, use of active learning methods	Direct education
School-based actions, media actions	Indirect training and control measures
Media, mobile and cyberspace	Distance learning
Care centers, attendance	In-person training

### Motivation analysis

Some students believed that substance use was a way to control and vent emotions and that smoking was a sedative.

Participant No. 9: "Drugs are substances that affect human emotions, such as crack, amphetamines, and cigarettes, which excite most young people”.

Participant No. 5: "When smoking, they may relax a little, but when they do not, they may get nervous."

The belief that using substances is not harmful was also evident in some students. In addition, some students also believed in the healing properties of substances.

Participant No. 6: "Some older men and grandfathers say that substances are like a cane for them, and they use it for leg pain”.

Participant No. 7: "Someone may have a cold, has a smoke, and gets well."

Students also considered imitation and curiosity as one the reasons for some people starting to use substances (Table 3).

**Table 3:** Categories and subcategories obtained from motivation analysis

Subcategories	Categories
Belief in the excitement of substance, belief in substance drunkenness, belief in the sedative of substance	Use substance to control and vent emotions
Avoid stimuli, delaying the response	Measures to prevent excitement
Excitement, calming	Manifestation and control of emotion
Belief in the harmlessness of substance, Belief in the healing properties of substance	False beliefs
Curiosity, imitation	Curiosity and imitation
Games and fun, sports activities, Remember God	Entertainment
Performing risky individual emotional activities, performing dangerous emotional activities in relation to other people	Dangerous solutions to vent emotions

### Opportunity analysis

Personal privacy and uncrowded public places were physical opportunities that students cited for substance use.

Being alone at home, being alone with friends, dilapidated houses, and buildings under construction were private places where students were likely to use more substances. Conversely, students believed that parks, neighborhoods where drugs are distributed, teahouses, and other public places are environments that people are more likely to engage in substance use.

According to the students, social opportunities for substance use included the role of family and friends and the role of the school. For example, imitating friends, family supervision, parental influence, older sibling influence, providing emotional outlets by parents, and the help of school counselors can all serve as social opportunities in substance use.

Students considered places such as libraries, homes, relaxation areas, mosques, sports clubs, villages, and backyards as suitable places to fill their leisure time and secluded places, alleys, and parents' workplaces as unsuitable places to spend their leisure time (Table 4).

**Table 4:** Categories and subcategories obtained from opportunity analysis

Subcategories	Categories
Solitude with friends, being alone somewhere, buildings under construction, ruins, addicted friend house	Privacy personal places
Parks, neighborhoods where drugs are distributed, teahouses, lonely places	Private public places
Imitate and accompany friends, family support and assistance	The role of family and friends in the manifestation of emotions
Assistance from school counselors	The role of the school in the proper filling of leisure time
Library, relaxing places, mosque, sport club, village, yard	Proper places in the proper filling of leisure time
Lonely places, alley, parents' workplace	Inappropriate places in the proper filling of leisure time

## Discussion

A focused group discussion was conducted to analyze the capability, motivation, and opportunity to prevent substance abuse in sensation-seeking junior high school boys on the outskirts of Birjand.

Based on the results, students' knowledge and understanding of the consequences of substance abuse was insufficient. Therefore, they did not understand the nature of substance abuse and its consequences. Although they named several common substances, they did not know all the substances and were not aware of all the dangerous side effects of substance abuse. This finding is consistent with the findings obtained in Alhyas et al. [16].

Unfortunately, some of the students found substance abuse beneficial to their health and to treat some ailments. They did not know enough about the underlying factors of substance abuse and were unaware of how people became addicted to substances. Adolescents living on the city's borders only find partial information about substances from those around them, and often this misinformation emphasizes the recreational aspects of substances. Students did not have the necessary skills to deal with stress, disconnect from an inappropriate friend, and adequately vent their emotions. This finding is consistent with the findings obtained in Javanmard [17].

Not all students had the right attitudes and beliefs about substances and their abuse. More importantly, some of them believed that the one-time use of substances was harmless. Some students saw substance use as a way to control their emotions and relax. This finding is inconsistent with Karimi and Zinivand's study results, which show that 91% of students have the right attitude towards substances [18]. The reason for this disparity can be the difference in the study population in Western Iran. Belief in the safety of one-time use of substances and curiosity about substances is extremely dangerous and can influence substance abuse. The results of this research were in line with the research by Jabbari Beyrami et al. that showed a significant proportion of the subjected studied specified curiosity as a factor for substance addiction [19]. Therefore, it is essential to pay attention to motivational factors related to substance abuse, and it can play a role in a students' tendency to use substances.

Concerning substance abuse, in addition to capacity and motivation, it is vital to pay attention to the opportunities facing students. For example, it can be part of parental control and supervision to preside over students in public and private places. Relationships with family, friends, classmate parents, and how to utilize leisure time are social opportunities related to substance abuse in students, and employing these opportunities influenced their tendency to substance abuse. This finding is consistent with the findings obtained in Meymandi et al. [20] and Yoosefi Lebni et al. [21]. Using the results of this study, more knowledge of students' educational needs for designing an addiction prevention educational intervention based on the COM-B model was gained in excited students, and these findings help to design a more effective educational intervention.

In order to prevent interventions against adolescents' tendency to substance abuse, it is important to pay attention to their capacities, motivations, and opportunities. In addition, to achieve more specific interventions that significantly impact their behaviors, it is beneficial to study the factors mentioned above before the intervention by conducting interviews or group discussions in some of the target individuals of the intended intervention. Better understanding of these factors and finding ways to change them can help to design more effective educational interventions to prevent the tendency to substance abuse in adolescents.

## Data Availability

The datasets used and analysed during the current study are available from the corresponding author on reasonable request.
